# What Technology Can and Cannot Do to Support Assessment of Non-cognitive Skills

**DOI:** 10.3389/fpsyg.2019.02168

**Published:** 2019-09-25

**Authors:** Vanessa R. Simmering, Lu Ou, Maria Bolsinova

**Affiliations:** ACTNext by ACT, Inc., Iowa City, IA, United States

**Keywords:** non-cognitive, competencies, assessment, construct validity, technological advances, theoretical limitations

## Abstract

Advances in technology hold great promise for expanding what assessments may achieve across domains. We focus on non-cognitive skills as our domain, but lessons can be extended to other domains for both the advantages and drawbacks of new technological approaches for different types of assessments. We first briefly review the limitations of traditional assessments of non-cognitive skills. Next, we discuss specific examples of technological advances, considering whether and how they can address such limitations, followed by remaining and new challenges introduced by incorporating technology into non-cognitive assessments. We conclude by noting that technology will not always improve assessments over traditional methods and that careful consideration must be given to the advantages and limitations of each type of assessment relative to the goals and needs of the assessor. The domain of non-cognitive assessments in particular remains limited by lack of agreement and clarity on some constructs and their relations to observable behavior (e.g., self-control versus -regulation versus -discipline), and until these theoretical limitations must be overcome to realize the full benefit of incorporating technology into assessments.

## Introduction

Non-cognitive skills have been increasingly recognized as important contributors to education and workplace success (Levin, 2013). These skills include a wide range of competencies, such as perseverance, collaboration, emotional intelligence, and self-regulation; Table 1 list those included in a recent systematic review (Smithers et al., 2018). There is some disagreement on how to define and delineate them, including whether such attributes are fixed traits or malleable skills (for discussion, see Lipnevich et al., 2013; Duckworth and Yeager, 2015; Smithers et al., 2018; Simmering et al., 2019). Although these are important theoretical issues that will inform assessment development, they are beyond the scope of the current paper. Rather, we discuss how advances in technology may change non-cognitive assessments. We aim to provide a high-level overview of advantages gained through technology, along with new and remaining challenges that must be addressed. We focus on non-cognitive skills because many are more contextual and dynamic than academic skills (e.g., delay of gratification, emotional reactivity). Before considering technological advances, we first briefly review the limitations of traditional non-cognitive assessments.

**Table 1:** Non-cognitive skills included in Smithers et al. (2018) systematic review and meta-analysis.

**High-level descriptors**
Character skills
Executive functions
Personality traits
Socio-emotional skills
Soft skills
**Specific capabilities**
Attention
Cognitive flexibility/control
Conscientiousness
Delay of gratification
Effortful control/self-control/regulation
Emotional stability/reactivity/regulation
Impulsivity
Inhibitory control
Locus of control
Motivation
Perseverance/persistence
Responsibility
Self-esteem
Sociability

*Smithers et al. did not differentiate terms as high-level versus specific; this has been added to acknowledge the multidimensional nature of the high-level constructs, though we recognize that some specific capabilities may also be multidimensional. We also group terms we viewed as synonymous within specific capabilities, although these views are not universal in the broader literature*.

## Common Limitations in Assessments of Non-Cognitive Skills

Duckworth and Yeager (2015) reviewed concerns with measurement of non-cognitive skills, outlining limitations of two types of assessments, questionnaires, and performance tasks, using the construct self-control for illustration (see Simmering et al., 2019, for related discussion). Questionnaires can be administered to any informant but most commonly use parent- and teacher-report for children and self-report for adolescents and adults. Questionnaires may ask about a subject’s behavior in general, in a specified period (e.g., at this moment, in the past week, month, or year), or in a hypothetical situation [as in situational judgment tests (SJTs)]. Responses may be ratings of frequency (e.g., “almost never” ranging to “almost always”), how well a description fits the subject (e.g., more or less true or like the individual), or choices of specific behaviors in SJTs. The limitations Duckworth and Yeager described were misinterpretation of items, lack of insight or information, insensitivity at different time scales, and reference or social desirability bias. Simmering et al. (2019) also noted context insensitivity as a limitation, as behaviors may occur in some contexts but not others that are not differentiated by questionnaires (e.g., perseverance in school work versus hobbies, or different academic subjects). Furthermore, some studies suggest that self-reports in response to hypothetical situations diverge from actual behavior in analogous experiences (Woodzicka and LaFrance, 2001; Bostyn et al., 2018). Limitations of questionnaires have been extensively studied (e.g., Furnham, 1986), with numerous remedies developed (e.g., Kronsik and Presser, 2009).

An alternative approach is to observe behavior directly rather than eliciting informants’ reflection and interpretation. Performance tasks are designed to compel behavior in relevant contexts, with the advantage of creating controlled situations in which all subjects are observed (for discussion, see Cronbach, 1970). For example, objective personality tests assess personality traits through behavioral indicators from performance tasks rather than self-reports (Ortner and Schmitt, 2014). Although performance tasks offer advantages over questionnaires – avoiding subjective judgments by informants, less opportunity for social desirability, reference, and acquiescence biases, more temporal sensitivity – they have serious limitations (see Duckworth and Yeager, 2015; Simmering et al., 2019, for further discussion). For example, lab-based performance tasks such as the Balloon Analogue Risk Task (Lejuez et al., 2002) typically assess single constructs (i.e., risk-taking) and lack diversity needed to form a complete personality profile. Performance tasks are generally designed to elicit one “right” behavior and may conflate “wrong” behaviors that reflect different underlying causes (e.g., Saxler, 2016). Participants’ behavior may reflect factors beyond the intended construct, such as compliance with authority of comprehension of instructions. This is a particular concern when participants’ prior experiences differ substantially from those designing, administering, and interpreting the tasks; behavior considered maladaptive in the task may be more appropriate to participants’ experience. Furthermore, task artificiality could create inauthentic motivations and constraints, leading to unnatural behaviors. Tasks with scenarios created in real time can also lead to error in task implementation, recording of behavior, or participant responses.

To overcome these types of limitations, Duckworth and Yeager (2015) recommended using multiple measures suited to the assessor’s goals while acknowledging and accounting for the limitations of each. They also noted that further innovation in assessment could avoid some limitations, with specific examples including incorporation of technology. In the next section, we review technological advances in non-cognitive assessments and the advantages they offer.

## Advantages of Technology-Enhanced Assessments

Technology allows new and expanded ways to collect data and present content. Computerizing assessments has become more common as access to technology has increased, but these implementations often merely reconfigure prior assessments to be presented on a screen without further adaptation. We focus on more substantive changes that expand the scope of the types of measurements and content included in non-cognitive assessments.

First, technology allows for real-time collection of multiple types of data, including self-reports, physiological data, and observed behavior. Traditionally, assessments are presented once or a few times at widely spaced intervals. Continuous, unobtrusive data collection is now possible through devices such as smartphones or fitness trackers. For example, Wang et al. (2014) combined multiple data sources from automated sensors on a smartphone (i.e., accelerometer, microphone, light sensing, global positioning, Bluetooth) with self-report sampling to evaluate how college students’ daily activity related to their mental well-being (i.e., depression, stress, loneliness) and academic performance. Sensor data correlated moderately with these outcomes, as well as students’ self-reports. These data were then used to infer students’ studying and social behavior to predict their GPA (Wang et al., 2015), indicating how sensor data could be used instead of self-reports. Automated sensors are not only less obtrusive to participants but can also provide a more temporally complete record, which avoids relying on narrow sampling and extrapolation to track change over time (c.f., Adolph et al., 2008). Such temporal detail is necessary to evaluate dynamic non-cognitive skills, such as self-regulation.

Second, ecologically valid methods allow data collection directly from relevant contexts, avoiding the need for retrospection or generalizations in questionnaires, imagined experiences in SJTs, or contrived scenarios in a lab (see Stone and Shiffman, 1994, for related discussion). Experience sampling methods, such as ecological momentary assessments and daily diaries, ask participants report thoughts, feelings, behaviors, and environment at regular intervals over time or around target events. They have been widely used to track emotions in natural contexts, allowing assessment of emotion regulation (Silk et al., 2003; Tan et al., 2012). When contextual variation is also recorded, these assessments can tally how frequently a subject encounters specific contexts and whether behavior varies across those contexts.

Third, some devices allow data collection not attainable without technology. For example, during computerized activities, participants’ eye movements can be continuously recorded using eye-trackers, and mouse movements or touchscreen selections can be collected using specialized software. Such data were inaccessible before technological solutions were developed, and they provide the opportunity for more holistic analysis of behavior. Assessments that provide these and other types of process data during participation, such as item-level response latencies (e.g., Ranger and Ortner, 2011), allow researchers to use more than just final responses to improve measurement. For example, pupillometry and reaction times can differentiate whether participants were controlling attention proactively (i.e., mentally preparing for target actions) versus reactively (i.e., adjusting action following external signals) even when target actions (i.e., identifying a stimulus sequence) did not differ (Chatham et al., 2009). Log files of online game-based assessments include time and event information that can be used to track participants’ collaboration during the game (Hao et al., 2016; Hao and Mislevy, 2018). Process data may provide insight into responses that would not be possible without technology, and analyzing such data can support assessment validation (Lee et al., 2019).

Beyond data collection, technology enables presentation of content in ways not possible with traditional assessments. Computerized adaptive testing draws items from a large pool of items with varying difficulty to present them adaptively based on test-takers’ previous responses and estimated ability (Segall, 2005). This allows more sensitivity to student ability levels and reduces the influence of small mistakes and lucky guesses on the final estimated ability. While computerized adaptive testing is most often used to measure cognitive abilities, it can also improve the measurement of other constructs, like personality (Makransky et al., 2013) and mental health (Becker et al., 2008; Stochl et al., 2016). Because adaptivity is an important facet of non-cognitive skills, test design and administration organizations such as the National Center for Education Statistics recommend adaptive tests in collaborative problem solving and other future assessments (Fiore et al., 2017).

Beyond contingent item presentation, interventions can also be integrated into computerized assessments. Based on assessment results, personalized feedback and recommended learning materials can be provided to respondents to improve individual development. Such systems have gained popularity in assessments of cognitive skills (e.g., Klinkenberg et al., 2011) but can also support non-cognitive skills. For example, Hutt et al. (2017) developed an eye-tracking application to monitor students’ mind-wandering in real time during a computerized learning task. When mind-wandering is detected, the application intervenes to repeat the recent material, redirect the student’s attention, or ask a question to allow self-reflection in the student. Although the goal was to improve students’ learning of the material, feedback on the frequency of mind-wandering could also teach students to monitor and regulate their mental engagement.

The nature of the material going into assessment items can also be expanded by technology. Rather than presenting text questionnaires, researchers can create multi-modal vignettes to present scenarios like SJTs. Audio-visual presentation is preferable to text for students with limited reading comprehension and can increase the validity for such groups (e.g., Chan and Schmitt, 1997). Through interactive technology like digital games and virtual or augmented reality, more complex content can be created to simulate real-life contexts that may be difficult to observe naturally. These environments can include “stealth” assessments in which students’ capabilities are evaluated without explicit queries. For example, in a role-playing game comprising quests that require creative problem solving, players’ actions may be scored for evidence of both cognitive (e.g., reading comprehension) and non-cognitive (e.g., persistence) competencies (Shute, 2011). Embedding target constructs in naturalistic interactions allows participants to respond with authentic behaviors rather than reporting imagined behavior in response to a hypothetical scenario. This can increase motivation and engagement when properly designed (Moreno-Ger et al., 2008), which in turn could reduce measurement error.

Technological advances can also facilitate generation of new content with reduced human effort, a vital feature for delivering assessments at scale. Machine learning and artificial intelligence have been developed for generating traditional assessment content (i.e., item stems and response options), although much work remains to achieve wide adoption (Gierl et al., 2012). One potential advantage to automated content generation, beyond the efficiency, is the expanded ability to personalize material for students. For example, research on motivation and engagement suggests that integrating students’ social and cultural identities into instructional and assessment design can improve outcomes for students from marginalized groups (Haslam, 2017). More work is needed to identify the best ways to design non-cognitive assessments to align with students’ identities, but technology provides a promising avenue to realize this level of personalization.

## Challenges in Adopting Technology-Enhanced Assessments

Technology-enhanced assessments are not without challenges and limitations. First, construct validity remains a significant concern, and adapting previous assessments to incorporate new technology may affect validity positively or negatively. As noted above, video vignettes in SJTs increased validity by decreasing the influence of reading comprehension (Chan and Schmitt, 1997). Conversely, more complex scenarios could introduce variation in interpretations or decision processes by participants. Such complexity likely reflects real-life contexts more closely but introduces challenges for standardization, especially when content presentation is contingent on participant performance. Standardized items and tasks, as well as scoring rubrics, for virtual performance assessments must be developed and validated in pilot studies (Hao et al., 2017).

The collection of more extensive, ecologically valid, and objective measures of behavior, whether during natural experience or games and simulations, still requires interpretation of how behaviors relate to underlying constructs (an important facet of construct validity; Borsboom et al., 2004). For example, although Hutt et al. (2017) related pupillometry and saccade duration to mind-wandering, these behaviors could be driven by external factors rather than internal processes. Similarly, data from automated sensors (as in Wang et al., 2014) cannot directly address whether variation in recorded activities reflects internal differences (i.e., participants’ self-regulation abilities) versus external forces. It is also possible that behaviors measured in these ways are not representative: knowing one is being observed in daily life may lead to atypical behavior, especially when a device is first introduced (c.f., Alvero and Austin, 2004), or participants may be more willing to act “out of character” in a simulation.

Second, one must consider both ethical issues shared with traditional assessments (e.g., how data will be stored, used, and potentially shared; proper training for those administering and interpreting assessments) and new issues that arise with technology. Technological requirements can contribute to inequity, as not all communities have access to necessary infrastructure (e.g., internet bandwidth, devices meeting specifications) or funding to adopt high-tech assessments, and participants may be unaccustomed to using technology. Automated or continuous recordings may invade the privacy of participants or non-participants who have not consented to have their data collected (e.g., conversation partners in audio recordings); although these concerns would be addressed through human subjects protections for research, such protections do not extend to assessments in non-research settings. Ethical concerns for developing technological assessments are conceptually similar to traditional assessments but may be practically different. For example, machine learning algorithms may be biased due to the training sets used to develop them (Springer et al., 2018) similar to how questionnaires may be biased by validation with unrepresentative samples (Clark and Watson, 2019).

Third, collection of more varied and continuous data introduces challenges in compliance and data management. Participants may find continuous or frequent sampling intrusive and therefore be less willing to complete an assessment. Imperfections in devices and software can lead to lost data, with some sources of loss relating to constructs of interest (e.g., losing track of eye gaze if posture changes as interest wanes). The multitude of possible reasons underlying data loss across different types of sensors and devices, combined with reasons shared with traditional assessments (e.g., selectively omitting responses, attrition), makes addressing missing data both practically and theoretically complex.

How we make use of more and different types of data across sources also presents new challenges. Connecting multiple assessments to the same individual profile requires complex data management solutions to ensure both privacy for individuals and accessibility for those using assessment results. If multiple sources are used simultaneously in real time, the data streams must be synchronized and at compatible granularity. Intensive longitudinal datasets require developing identifiable statistical models that can accommodate irregularly spaced, high-dimensional, noisy, dynamic data, as well as related robust and efficient computing software to make use of them (Chow et al., 2018).

Lastly, there can be a strong temptation to apply new technology to assessment as it becomes available without fully evaluating the potential costs and benefits of its adoption. It is important not to let technological capabilities be the driving factors in assessment development but rather to focus on the need the assessment is serving and whether that need can be better met by technology. New technological applications must be carefully designed and validated even when they seem to be only a minor change from previous methods. For example, moving from text to audio-visual presentation of SJTs introduces decisions for how each character looks and sounds. Participants may interpret or respond to characters’ behavior differently based on demographic features (c.f., Renno and Shutts, 2015) or voice intonation, which can unintentionally alter the content from the text version. Each new development will bring in new considerations for how the method reaches assessment goals.

## Conclusion

Advances in technology have expanded the horizons of what types of assessments are possible and achievable. These expansions can contribute to our understanding of non-cognitive capabilities as well as traditional academic content. The advantages of technology-enhanced assessments include how and what data can be collected, as well as the content that can be presented. With these advantages come some new challenges in the implementation and analysis of assessments, as well as the familiar challenges of construct and predictive validity that all assessments must address. Whether technology can improve an assessment will depend on details of the construct, the target group, the aims of the assessment, and the desired implementation. Assessment methods should be tailored to the specific conditions at hand. In the context of non-cognitive assessments in particular, more work is needed to arrive at well-defined constructs with clear connections to behavior as we also work to capitalize on the advantages technology offers.

## Author Contributions

VS conceptualized the topic and all three authors contributed equally to development of the ideas. VS drafted the manuscript, then LO and MB provided critical additions and revisions.

### Conflict of Interest

VS, LO, and MB were employed by the non-profit company ACT, Inc.
